# Preventing microbe colonization on avocado (*Persea nubigena* var. *guatemalensis*) through metabiotic treatment, a promising postharvest safety improvement

**DOI:** 10.3389/fmicb.2024.1344735

**Published:** 2024-03-13

**Authors:** Gabriela N. Tenea, Evelyn Angamarca, Victor Cifuentes, Jazmin Hidalgo

**Affiliations:** Biofood and Nutraceutics Research and Development Group, Faculty of Engineering in Agricultural and Environmental Sciences, Universidad Técnica del Norte, Ibarra, Ecuador

**Keywords:** metabiotics, antimicrobials, avocado, *Staphylococcus*, fruits bioprotectors

## Abstract

**Introduction:**

Lactic acid bacteria (LAB) produce various metabolites (i.e. metabiotics) with inhibitory capacity towards harmful foodborne pathogens.

**Methods:**

This study aimed to design several antimicrobial formulations based on metabiotics obtained from different native LAB species (*Lactobacillus pentosus* UTNGt5, *Lactococcus lactis* UTNGt28, and *Weissella cibaria* UTNGt21O) and to detect the possible mode of action towards two multidrug resistant *Staphylococcus* spp. strains isolated from avocado (*Persea nubigena* var. *guatemalensis*) fruits. Additionally, the formulation with the highest inhibitory activity was tested *ex vitro* on avocados at the immature (firm) ripeness stage to evaluate their effect on microorganisms’ growth and fruit quality attributes post-harvest.

**Results and discussion:**

Out of the top five formulations showing the highest bactericidal effect *in vitro* at their minimum inhibitory concentration (1 x MIC) on both *Staphylococcus* spp. targets one candidate annotated P11 (consisting of UTNGt21O and UTNGt28; 1:3, v/v) was selected. Co-cultivation of *Staphylococcus* strains with P11 formulation results in cell viability reduction by 98%, by impairing the integrity of the cell membrane inducing cytoplasm molecule content leakage, protein profile changes, and finally bacterial death. Even though the total coliforms, *Staphylococcus* spp., *Enterobacte*r spp., molds, and yeasts counts were not fully eliminated by day 13 of storage, a statistically significant reduction (*p* < 0.05) in viable cell counts were observed by day 8 upon the P11 treatment compared with non-treated control (C) and treated with a commercial disinfectant (T1) samples, suggesting that P11 formulation inhibited microbial colonization during storage. Likewise, no visible dark spots were observed on the mesocarp (pulp) upon the treatment with P11, whereas T1 and C fruits showed greater dark spots on the pulp as indicative of damage. The quality attributes, such as pH, total soluble solids, total titratable acidity, antioxidant capacity, and total polyphenol content, were not affected by the treatment. Principal Component Analysis (PCA) conducted on these five variables showed a clear separation of samples according to the maturity stage regardless of the treatment.

**Conclusion:**

These results suggest that the active metabolites from LAB strains might create a barrier between the exocarp and mesocarp, inhibiting the microorganisms colonization, reducing fruit damage, and lengthening the fruit quality and safety after harvest.

## Introduction

Avocado is a common crop in Ecuador (Álvarez Flores et al., 2021). The Fuerte variety (*Persea nubigena* var. *guatemalensis*) accounts for 99% of domestic consumption, and the Hass variety (*Persea americana* ‘Hass’) is mainly exported. Avocado production and marketing are new ways of boosting the economy since local market prices are accessible. A kilogram of avocados costs approximately USD 0.60, while it costs three times as much in Europe. The avocados from the Andes and the Ecuadorian coast are non-traditional products that help thousands of farmers in rural areas by creating jobs, wealth, and opportunities to enhance their quality of life (Álvarez Flores et al., 2021). This crop also enables changes in rural landscapes and crop diversification, making it strategic in the medium and long term for an important industrial sector (MAGAP, 2021). Compared with Hass, the cultivar Fuerte is much more perishable (García-Frutos et al., 2020). Preharvest factors, harvest time, and postharvest practices affect fruit quality. These fruits are harvested at the immature, firm ripeness stage, which is known as bright green fruit that is ready to eat in four days. Poor handling and storage practices can increase the likelihood of postharvest bacterial contamination because the fruit’s outer peel is thicker and low-frost resistant. Several studies have revealed the presence of pathogens in the Hass variety (García-Frutos et al., 2020; Aliero et al., 2022). On the other hand, fungicide treatments are mostly used to stop the process of fruit deterioration, but they have been shown to interfere negatively with human health (Munhuweyi et al., 2020). As consumer awareness of the negative effects of fungicide use has increased during the past 10 years or more, numerous research initiatives have been established to discover safer alternative technologies (Ssemugabo et al., 2023). Physical heat treatments (like hot water dips, steaming, dry heat, and forced air), irradiation, controlled atmospheres, biological control, plant extracts (like essential oils), and elicitors (like chitosan and jasmonates), among others, can be categorized among these alternative techniques (Tesfay and Magwaza, 2017; Romanazzi et al., 2018). Several efforts have been centered on searching for natural and safe products for fruits and vegetables for postharvest decay protection (Agriopoulou et al., 2020). Nonetheless, few biocontrol products are commercially available, but they have short market lifetimes and are not accessible to all countries (Poleatewich et al., 2023). Despite government efforts, food safety initiatives in Ecuador are still difficult to implement. The information regarding quality standards is limited. Improper manipulation, mechanical damage, and inappropriate storage after harvest are among the common factors that affect the bacteriological quality of these crops. Recently, using metagenomics and conventional bacteriological analyses, we showed that the Fuerte variety harbors multi-drug-resistant bacteria such as *Enterobacter* spp. and *Staphylococcus* spp., in both the immature and mature stages (Angamarca et al., 2023). To achieve an increase in the production and marketing of Fuerte cultivars in Ecuador, it is vitally important to consider valuable strategies to protect the fruit after harvest. The key to prolonging the shelf life of avocados in a risk-free and health-conscious way is to develop postharvest management strategies employing environmentally friendly and non-chemical approaches.

The use of LAB and their metabolites (i.e., metabiotics) as an alternative for natural food preservation is seen to be a very promising method for maintaining the microbiological purity and safety of postharvest storage of raw and little processed fruits and vegetables, both alone and in combination with edible coatings (Biswas and Das Mohapatra, 2023; Islam et al., 2023). These bacteria are known as food grade and are used as preservatives to stop the growth of pathogens and as potential antibiotic alternatives to prevent or treat a variety of foods (Terpou et al., 2019). These active molecules produced by probiotics are also harmless and have no negative impact on human health (Vieco-Saiz et al., 2019). So far, only nisin obtained from *Lactococcus lactis*, pediocin PA-1/AcH obtained from *Pediococcus acidilactici*, and Micocin® obtained from *Carnobacterium* spp. have received FDA approval for use as food additives (FDA American Food Drug Administration, 2016). Despite several studies having assessed their usage in fruits and vegetables as alternatives to artificial food preservatives *in vitro*, their application on an industrial scale is reduced (Barbosa et al., 2013; Bahrami et al., 2020). Nonetheless, the antimicrobial effect of LAB-producing molecules has mainly been investigated *in vitro* toward several artificially inoculated pathogens (Siroli et al., 2015; Tumbarski et al., 2019; Vieira et al., 2019), but fewer studies are focusing on the native microbiota that colonize the fruits after harvest. In previous research, we selected several LAB strains that produce metabolites with antimicrobial activity. Among them, some species from *Lactobacillus, Lactococcus,* and *Weissella* genera produce metabolites with high inhibitory potential. Moreover, we designed some formulations based on peptide-protein extracts (PPE) from these LAB strains and tested their effect on tomatoes (Tenea and Pozo Delgado, 2019). In addition, their whole genome was sequenced, and genes involved in antimicrobial action were annotated (Tenea and Hurtado, 2021). Considering that the inhibitory effect varies with the species antimicrobial strength as well as depends on the food matrix characteristics are intended to be applied, the current study aimed to design several PPE-based formulations using combinations of metabiotics obtained from three LAB strains, screen for their antimicrobial activity *in vitro* against two native avocado multi-drug-resistant *Staphylococcus* strains (FFCShyA2 and FFCShyA4), and detect their possible mode of action. Moreover, the most effective formulation was tested *ex vitro* on avocado fruits to evaluate their ability to inhibit the colonization of microorganisms on fruit surfaces after harvest. Finally, the effect of PPE-based formulations on physicochemical (pH, total soluble solids, total titratable acidity) and functional (total polyphenol content, antioxidant capacity) attributes were assessed.

## Materials and methods

### Bacterial strains

*Lactobacillus pentosus* UTNGt5 (GenBank Accession No. ON307470), *W. cibaria* UTNGt21O (GenBank Genome Assembly SRX8614718), and *L. lactis* strain UTNGt28 (GenBank Accession No. MG675576.1) were previously isolated from wild tropical fruits from the Amazon Forest (Tenea and Hurtado, 2021). Stocks of these strains are kept at the CCMBIOGEM Microorganisms Collection (Microbial Biotechnology Research and Development Laboratory-BIOGEM, Universidad Tecnica del Norte) and are available for research purposes upon request. Fresh cultures were obtained by cultivation on MRS (Man, Rogosa, and Sharpe) agar (Difco, USA) at 37°C before use. Two target multi-drug-resistant indicator bacteria, *S. xylosus* FFCShyA2 (GenBank Accession No. OQ372998.1) and *S. saprophyticus* FFCShyA4 (GenBank Accession No. OQ373001.1), previously isolated from the Fuerte variety of avocado were used. These target bacteria were grown in BHI (Brain Heart Infusion, Merck Millipore, MA, USA) broth media. All microorganisms were maintained at −80°C in 20% glycerol (*v/v*).

### Establishment of PPE-based formulations, determination of minimum inhibitory concentration, and antimicrobial capacity against *Staphylococcus* strains

Overnight UTNGt5, UTNGt21O, and UTNGt28 cultures (MRS-broth, 37 °C) were used to extract cell-free supernatant (CFS) by centrifugation at 13,000 × *g* for 20 min (4°C) followed by filtration using a 0.22 μm porosity syringe filter (# STF020025H, Chemlab Group, Washington, DC, USA). CFS was precipitated with ethyl acetate (v/v), followed by 24 h incubation at a low temperature without stirring, and centrifuged for 30 min at 8000 × *g*. The PPEs were recovered in 25 mM ammonium acetate (pH 6.5), desalted using a midi dialysis kit (# PURD10005-1KT, Sigma-Aldrich Co. LLC, Saint Louis, MO, USA), pre-equilibrated with phosphate buffer (pH 7.0), dried for 48 h under the flow chamber, recuperated in sterile water, and stored at –20°C. Supplementary Table S1 described the established PPE-based formulations evaluated in this study against FFCShyA2 and FFCShyA4 strains *in vitro,* using the agar-well diffusion method. Titer, estimated as AU/ml (defined as the highest dilution that inhibited the growth of the indicator strain), was determined (Ge et al., 2016). Each established formulation with a determined concentration (ranging from 200 to 9,800 AU/ml) was added independently into broth tubes containing the target bacteria and incubated for 24 h at 37°C, followed by plate agar to determine the minimum inhibitory concentration (MIC) that reduced the bacterial growth by 90% (Yasir et al., 2019). The titer for FFCShyA2 and FFCShyA4 was estimated at 800 and 1,600 AU/ml, respectively.

### Co-culture time-killing assay

To evaluate the effect of PPE-based formulations showing the highest antimicrobial activity according to the results obtained in the above-mentioned assay, as a function of time, we carried out co-culture time-kill experiments. The time-killing assay was performed as previously described (Wang et al., 2022). In brief, overnight FFCShyA2 and FFCShyA4 cultures (1 × 10^6^ CFU/ml) were inoculated independently with the selected PPEs at the 1 × MIC concentration and incubated at 37°C. As a control, untreated cells and treated cells with individual PPE (P2 and P3) were used. The cell viability was determined at different time intervals (0, 1, 3, and 6 h) using the plate-agar method (BD Difco plate count agar, Fisher Scientific Co. LLC, Hampton, NH, USA). All experiments were performed in triplicate.

### Leakage of aromatic molecules and proteininc profile assessment

The effect of the selected PPE-based formulations on both target bacteria cell integrity and proteinic profile was determined as described (Patra et al., 2015). In brief, overnight bacterial suspensions of each target (1 × 10^5^ CFU/ml) grown in BHI broth were washed twice with 1 × PBS (phosphate-buffered saline, pH 7.5) and treated independently with the selected PPEs for 24 h at 37°C. Bacterial cell culture without PPE treatment was used as the control. The release of DNA/RNA molecules was detected by electrophoresis in a 1% agarose gel with ethidium bromide, running in 1 × TBE (Tris-borate, EDTA, pH 8.0) buffer (Sigma-Aldrich Co. LLC, Saint Louis, MO, USA) after extraction with chloroform (1:1, *v/v*), and precipitated with isopropanol and ammonium acetate (3 M). The protein profile was analyzed using the Tricine-SDS-PAGE method. The remaining cell pellet after the incubation of both target pathogens with PPEs was suspended in 1 x SDS-PAGE loading buffer, boiled for 5 min at 100°C, and centrifuged at 300 x rpm. RunBlue Bis-Tris protein gels (12%) and Dual Cool Mini vertical PAGE/blotting Systems (Expedeon, Abcam, Cambridge, MA, USA) were used to visualize the protein profile of treated and untreated cells extract. The gel was stained with InstantBlue ready-to-use stain (Expedeon, Abcam, Cambridge, MA, USA) using a protocol recommended by the manufacturer.

### Effect of P11 on avocado fruits during shelf-life

#### Phenotypic evaluation

The color and status change from bright green, underripe to dark green, ripe; the visible black spots and fungi forming on the fruit surface and the pulp aspect were evaluated during 13 days of storage. The avocado fruits (five fruits per treatment, total 15 fruits x three repetitions = 45 fruits) at the immature ripeness stage (firm bright green, underripe) with no visible damages were purchased by a local retail vendor (Ibarra city), washed with 5% bleach solution for 5 min then twice with tap water and twice with distillate water, and left to dry under a biosafety cabinet. The fruits were immersed in (a) the P11 solution, (b) commercial disinfectant (Star Bac Domestic, a bactericidal solution) prepared according to the manufacturer’s instructions (T1), and (c) sterile water (C) in a final volume of 200 ml for 15 min. The fruits were left to dry, transferred to paper trays, and stored at room temperature in dark conditions.

#### Bacteriological analysis

In parallel, bacteriological analyses were performed on days 0, 1, 4, 8, 11, and 13, as described (Angamarca et al., 2023). Briefly, five fruits/treatments were placed independently in a Ziplock bag containing peptone water (1%) and incubated for 2 h at 37°C. The cells were recuperated by centrifugation for five min at 8000 × *g* and suspended in 1 × PBS (10 ml). The presence of target microorganisms was assessed in both 3 M Petrifilm and selective chromogenic media (Tenea et al., 2023). Blood agar and Brilliance Staph 24 Agar Medium (Oxoid Limited, Wade Road, Basingstoke, Hampshire, UK) were used to detect and enumerate *Staphylococcus* spp. (ISO 6888-1:1999/Amd 2:2018, n.d.). Independent experiment aliquots (100 μl) were placed on Chromocult Coliform agar (Merck Millipore, Kenilworth, NJ, USA) to determine the total coliforms and possible presence of *Escherichia coli* and eosin methylene blue (Difco, Detroit, MI, USA) to detect both *Enterobacter* spp. and *E. coli*. Detection and enumeration of yeasts and molds was performed with Dichloran Rose-Bengal Chloramphenicol (DRBC) Agar Base (Thermo Scientific™ Oxoid™, USA) after 7 days of incubation at 25–28°C. The experiments were run in triplicate and the microbial counts were expressed as CFU/g.

#### Determination of pH, total titratable acidity, and total soluble solids

The pH was determined using an electrode immersion pH meter (S210, Mettler Toledo, Columbus, OH, USA). Using phenolphthalein as an indicator, the total titratable acidity was measured during storage (days 0, 1, 4, 8, 11, and 13) by titrating 25 mL of pulp juice obtained with 0.1 N NaOH (AOAC, 2003). Results were expressed as a percentage of tartaric acid per 100 mL of juice. Total soluble solids content was determined using a digital refractometer (AOAC, 2003). Each experiment was carried out three times, using different batches of raw material.

#### Total polyphenol content estimation

The Folin–Ciocalteu method with gallic acid (Sigma-Aldrich Co. LLC, Saint Louis, MO, USA) as standard was used as previously described (Lyu et al., 2023). The pulp (5 g) of control (untreated), commercial disinfectant (T1), and P11 formulation was extracted with 20 ml of 80% (*v*/*v*) ethanol. Samples were centrifuged at 8000 × *g* at 4°C for 20 min in a benchtop centrifuge (Zentrifugen Rotina 380R, Hettich, Germany). The supernatants were filtered through a 0.45 μM hydrophilic filter (ANPEL Scientific Instrument Co., Shanghai, China) and used as extract (500 μl) for determining the total polyphenol content during storage (day 0, 1, 4, 8, 11, and 13). Absorbance at 715 nm was measured using a spectrophotometer (Jenway 6,705 UV / Vis, Bibby Scientific Limited, ST15 OSA, UK), and the graphical dependence of solution absorbance on the amount of gallic acid was plotted. The calibration curve was prepared with gallic acid standard (0–200 μg/ml), and the total polyphenol content result was expressed as mg of gallic acid equivalents (GAE) per gram (mg GAE/g) of the sample. The analyses were carried out in triplicate, starting with a new batch of samples (3 extracts).

#### Antioxidant activity determination

The DPPH (1, 1-diphenyl-2-picryl-hydrazyl, Sigma-Aldrich Co. LLC, Saint Louis, MO, USA) radical scavenging activity was determined during storage (days 0, 1, 4, 8, 11, and 13) as described (Fan et al., 2022). The supernatant was collected after treatment of each sample extract (5 mL) with ethanol 98% (10 mL) and centrifuged at 8000 × *g* for 20 min. Hundred μL of extract from control (untreated), T1, and P11-treated avocado was mixed with 2.9 mL of methanolic solution of DPPH (0.045 mg/ml). Absorbance was measured at 517 nm using an ultraviolet spectrophotometer (Jenway 6,705 UV/Vis, Bibby Scientific Limited, ST15 OSA, UK) after the mixture was kept in the dark for 30 min. The relative antioxidant in a mixture sample to scavenge DPPH was compared with a Trolox standard (Sigma-Aldrich Co., LLC, Saint Louis, MO, USA). The results were expressed in equivalent μmol Trolox/g fruit. The analyses were carried out in triplicate, starting with a new batch of samples (three extracts).

### Statistical analysis

The results were reported as mean ± standard deviation. To find significant differences between the means, the Kruskal–Wallis one-way analysis of variance (non-parametric) and Tukey’s *post hoc* test were used (SPSS version 10.0.6, US). *p* < 0.05 was selected as the statistical significance level (SPSS version 10.0.6, US). Moreover, the PCA of five variables (pH, total soluble solids, total titratable acidity, antioxidant capacity, total polyphenol content) on treated and untreated fruits with the PPE-based formulations were analyzed. Additionally, Pearson correlation was employed to determine whether the response variables interacted with each other.

## Results and discussion

### PPE-based formulations inhibit multi-drug-resistant *Staphylococcus* strains

**Figure 1:**
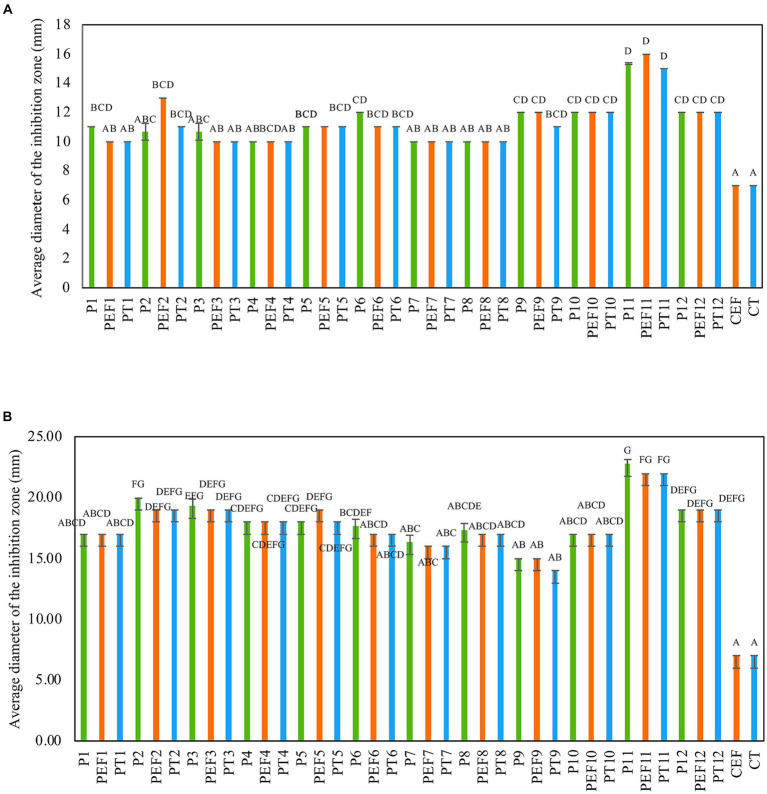
Antimicrobial activity of PPE-based formulations against **(A)** FFCShyA2 and **(B)** FFCShy4. The capital letters above the bars indicate significance ranges obtained by Kruskal–Wallis (*p* < 0.001). Green bars: individual PPEs and combination thereof (1 x MIC); Blue bars: (individual PPEs and combination thereof) + EDTA (0.1 mg/ml); Orange bars: (individual PPEs and combination thereof) + Tween 20 (1 mg/ml). P1: UTNGt5; P2: UTNGt21O; P3: UTNGt28; P4: UTNGt5 + UTNGt21O (1:1, v/v); P5: UTNGt5 + UTNGt21O (1:3, v/v); P6: UTNGt5 + UTNGt21O (3:1, v/v); P7: UTNGt5 + UTNGt28 (1:1, v/v); P8: UTNGt5 + UTNGt28 (1:3, v/v); P9: UTNGt5 + UTNGt28 (3:1, v/v); P10: UTNGt21O + UTNGt21O (1:1, v/v); P11: UTNGt21O + UTNGt28 (1:3, v/v); P12: UTNGt21O + UTNGt28 (3:1, v/v); PEF1: UTNGt5 + EDTA; PEF2: UTNGt21O + EDTA; PEF3: UTNGt28 + EDTA; PEF4: UTNGt5 + UTNGt21O (1:1, v/v) + EDTA; PEF5: UTNGt5 + UTNGt21O (1:3, v/v) + EDTA; PEF6: UTNGt5 + UTNGt21O (3:1, v/v) + EDTA; PEF7: UTNGt5 + UTNGt28 (1:1, v/v) + EDTA; PEF8: UTNGt5 + UTNGt28 (1:3, v/v) + EDTA; PEF9: UTNGt5 + UTNGt28 (3:1, v/v) + EDTA; PEF10: UTNGt21O + UTNGt28 (1:1, v/v) + EDTA; PEF11: UTNGt21O + UTNGt28 (1:3, v/v) + EDTA; PEF12: UTNGt21O + UTNGt28 (3:1, v/v) + EDTA; PT1: UTNGt5 + Tween 20; PT2: UTNGt21O + Tween 20; PT3: UTNGt28 + Tween 20; PT4: UTNGt5 + UTNGt21O (1:1, v/v) + Tween 20; PT5: UTNGt5 + UTNGt21O (1:3, v/v) + Tween 20; PT6: UTNGt5 + UTNGt21O (3:1, v/v) + Tween 20; PT7- UTNGt5 + UTNGt28 (1:1, v/v) + Tween 20; PT8: UTNGt5 + UTNGt28 (1:3, v/v) + Tween 20; PT9: UTNGt5 + UTNGt28 (3:1, v/v) + Tween 20; PT10: UTNGt21O + UTNGt28 (1:1, v/v) + Tween 20; PT11: UTNGt21O + UTNGt28 (1:3, v/v) + Tween 20; PT12: UTNGt21O + UTNGt28 (3:1, v/v) + Tween 20; CEF: EDTA; CT- Tween 20.

The antimicrobial activity of several formulations consisting of a combination of PPEs extracted from three LAB strains with and without the addition of a chelate agent such as ethylenediaminetetraacetic acid (EDTA) and polysorbate 20 (Tween 20) against two multi-drug-resistant *Staphylococcus* strains was assessed. Although all tested formulations (38) exhibited antibacterial activity, the annotated formulations P11(UTNGt21O + UTNGt28, 1:3, v/v), PT11 (UTNGt21O + UTNGt28, 1:3, v/v + Tween 20, 1 mg/mL), and PEF11(UTNGt21O + UTNGt28, 1:3, v/v + EDTA, 0.1 mg/mL) were the most effective *in vitro* (Figures 1A,B). Individual extracts, P2 (PPE extract from UTNGt21O strain) and P3 (PPE extract from UTNGt28 strain) showed marginal inhibitory effect (diameter of inhibition zone of 10.67 ± 0.55 mm) toward both target strains. EDTA and Tween 20 alone showed no activity. Additionally, when combined with PPEs, EDTA and Tween 20 enhanced the antimicrobial effect. The inhibition zone formed by selected formulations is shown in Supplementary Figure S1. The overall inhibitory effect was strain-, dosage-, and target-specific; however, these substances became effective at lower concentrations when antimicrobial combinations were used. A recent screening analysis of numerous LAB strains obtained from various food matrices revealed the selectivity of the strains against three skin commensals, *S. epidermis, S. hominis*, and *S. aureus* targets (Christensen et al., 2021). Depending on the strain’s susceptibility, chelating chemicals can stimulate inhibitory action and increase their spectrum (Juda et al., 2008). The Food and Agriculture Organization (FAO 21.CFR.172.120, 2020) recognizes EDTA and Tween 20 as food additives. Furthermore, Tween 20, a nonionic surfactant, may boost antimicrobial activity *in vitro*, whereas EDTA is an outer membrane permeabilizer (Alakomi et al., 2006; Gomez-Lopez et al., 2006). In this study, the inhibitory activity was target-dependent, which is in agreement with early research (Ghanbari et al., 2013). Furthermore, there may be a synergistic effect between the PPEs + EDTA or PPEs + Tween 20 as no effect was seen when both substances were used alone. Based on these results, we selected the P11 as the optimum inhibitory formulation for further investigation.

### P11 formulation diminishes *Staphylococcus* cell growth in co-culture

**Figure 2:**
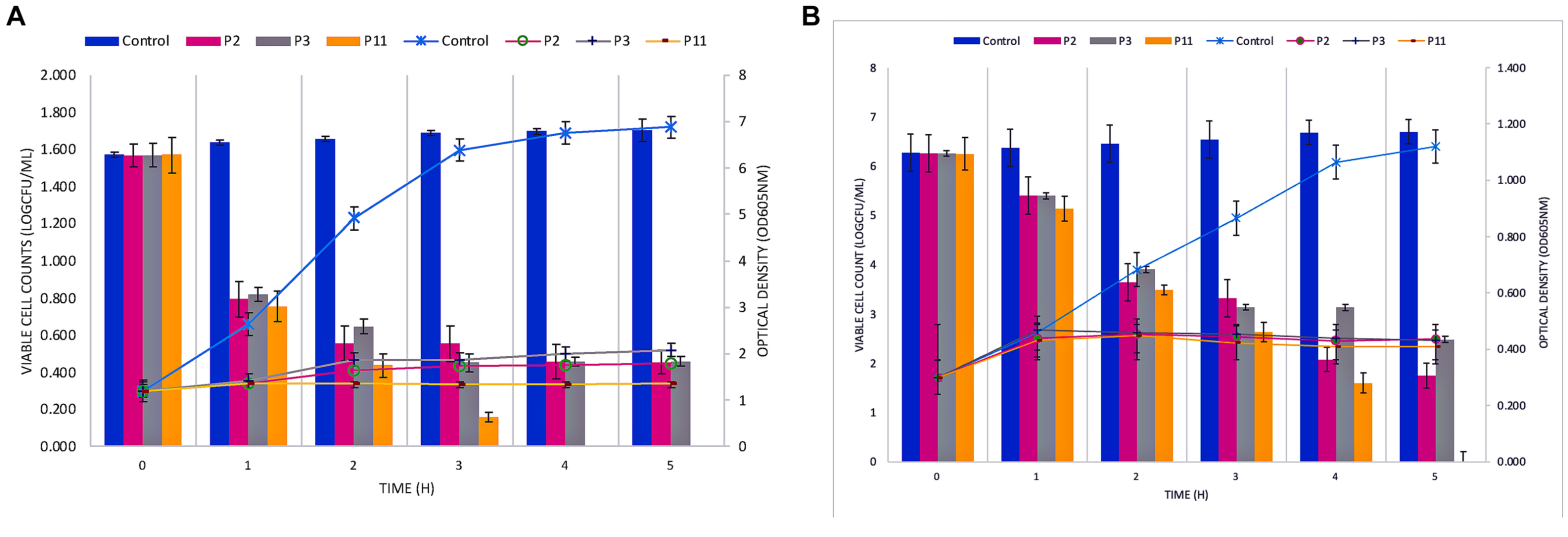
Co-culture of PPEs with *Staphylococcus* FFCShyA2 **(A)** and *Staphylococcus* FFCShyA4 **(B)** over time. Error bars represent the standard deviations of three replicates (*n* = 3). P2: (1 x MIC) UTNGt21O; P3: (1 x MIC) UTNGt28; P11: (1 x MIC) UTNGt21O + UTNGt28 (1:3, v/v); UTNGt21O: metabiotics from *W. cibaria* strain UTNGt21O; UTNGt28: metabiotics from *L. lactis* strain UTNGt28.

The bactericidal effect of the P11 on FFCShyA2 and FFCShyA4 was determined during 5 h in a co-culture assay (Figure 2). The co-cultures with P11 showed a significant reduction (*p* < 0.001) in viable FFCShyA2 cell counts by 3.0 log CFU/mL after 1 h of incubation with no viable cells detected after 4 h, while individual peptides P2 and P3 gradually decreased FFCShyA2 viable cells with the incubation time (Figure 2A). Similarly, P11 significantly reduced (*p* < 0.001) the viability of FFCShyA4 after 2 h of incubation with total cell viability loss registered after 5 h (Figure 2B). From the whole genome annotation analysis, we showed that the UTNGt21O strain (P2 producer) harbors a putative bacteriocin, with 33.4% sequence similarity to enterolysin A and a bacteriolytic effect toward *Salmonella* and *E. coli* at both the early and later logarithmic phases of growth (Tenea and Hurtado, 2021). This protein of 17kDa was not found in other *W. cibaria* strains retrieved from the National Centre for Biotechnological Information database. The UTNGt28 (P3 producer) harbor genes encode for two-peptide system lacticin 3,147, two-peptide lactococcin M (Class IIc), and Lactococcus-specific bacteriocin lactococcin A (Class IId). Plantaricins EF and JK significantly lyse *S. epidermidis*, as demonstrated by Selegård et al. (2019), and subsequent research by Musa et al. (2021) demonstrated how the two-peptide Plantaricin NC8 abolished *S. aureus* while reducing its inflammatory and cytotoxic effects. Taken together, a combination of metabolites produced by different LAB species displayed a bacteriolytic mode of action toward both target strains. Furthermore, it would be compulsory to determine which metabolite from the formulation alters cell growth.

### P11 formulation compromises membrane integrity and alters the proteinic pattern of target cells

**Figure 3:**
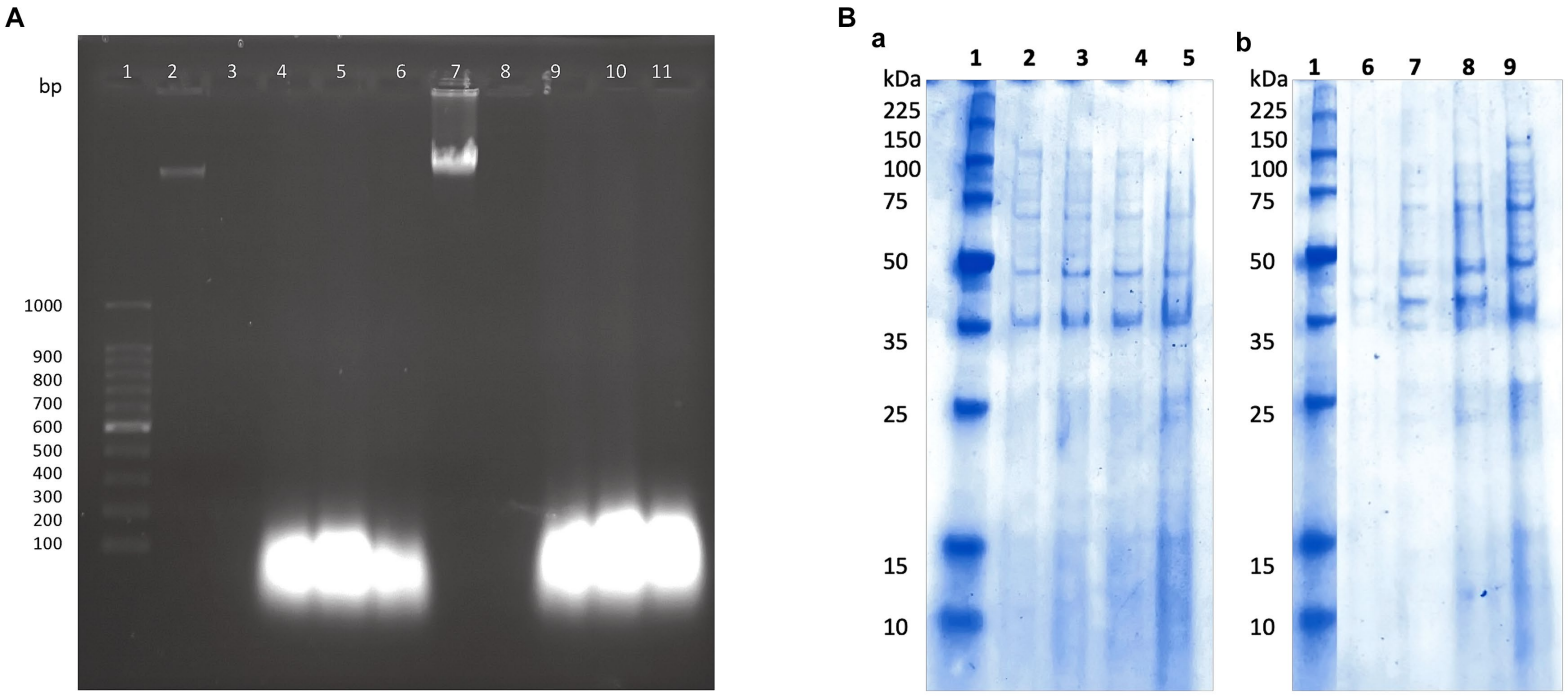
(A) The effect of P11 formulation on cell membrane integrity. **(A)** 1, 100 bp molecular marker; 2, 7: genomic DNA of FFCShyA2 and FFCShyA4; 3, 8: untreated target cells; 4–6 and 9–11: cell treated with P2, P3, and P11. **(B)** Different protein profiles upon the treatment of **(a)**. *S. xylosus* FFCShyA2 and **(b)**. *S. saprophyticus* FFCShyA4 with P11. 1: Broad range protein molecular weight marker (Promega #V8491). 2: *S. xylosus* FFCShyA2 untreated; 3: *S. xylosus* FFCShyA2 + P2; 4: *S. xylosus* FFCShyA2 + P3; 5: *S. xylosus* FFCShyA2 + P11; 6: *S. saprophyticus* FFCShyA4 untreated; 7: *S. saprophyticus* FFCShyA4 + P2; 8*: S. saprophyticus* FFCShyA4 + P3; 9*: S. saprophyticus* FFCShyA4 + P11; UTNGt21O: metabiotics from *W. cibaria* strain UTNGt21O; UTNGt28: metabiotics from *L. lactis* strain UTNGt28.

Previously, we found that the peptide extract from the UTNGt21O strain caused cell damage and the leakage of cytoplasmic molecules from gram-negative target bacteria (Tenea and Hurtado, 2021). Likewise, only RNA molecules were released when *S. aureus* ATCC1026 cells were incubated with different doses of the peptide extract, indicating that the *Staphylococcus* cell membrane was susceptible; however, cell death occurred at 24 h. Following the same pattern as seen with *S. aureus*, in this study, only RNA molecules were detected in agarose gel after the incubation of both target strains with the P11 formulation (Figure 3A). Thus, we suggest that after the interaction of peptide-protein extract with the cell membrane, the free DNA molecules are broken down, while RNA molecules might be protected by another molecular process. However, more research is required to confirm this statement. Cell integrity was not affected in the untreated control samples; thus, no DNA or RNA was detected in agarose gels. Complementary analysis of the proteinic profile upon treatment with the P11 formulation showed marginal changes in protein pattern in FFCShyA2 (Figure 3Ba). Visible changes in the proteinic profile were observed when FFCShyA4 was treated with P11, suggesting that this clone was more sensitive, showing both low- and high-weight proteins in the polyacrylamide electrophoresis gel (Figure 3Bb). According to early research (Xue et al., 2016), the interaction between the peptide and proteins with the target bacterium resulted in protein (lower and higher molecular weights) expression blockage. Additionally, when MPX (mastoparan 14-amino-acid peptide) was combined with *S. aureus* ATCC25923, the protein content increased in comparison to the untreated cell, suggesting that peptides may destroy the cell membranes, releasing high concentrations of proteins (Zhu et al., 2022). These outcomes were consistent with our prior findings that some peptides may cause gaps in the membrane proteins and visible phenotypic alterations in the target whole protein pattern (Tenea and Hurtado, 2021). P11 formulation, therefore, caused a bactericidal action *in vitro* by increasing membrane permeability, causing a loss of cellular integrity, leaking aromatic molecules, and releasing low- and high-weight molecular proteins.

### P11 formulation prevents microorganism colonization on avocado fruits

**Figure 4:**
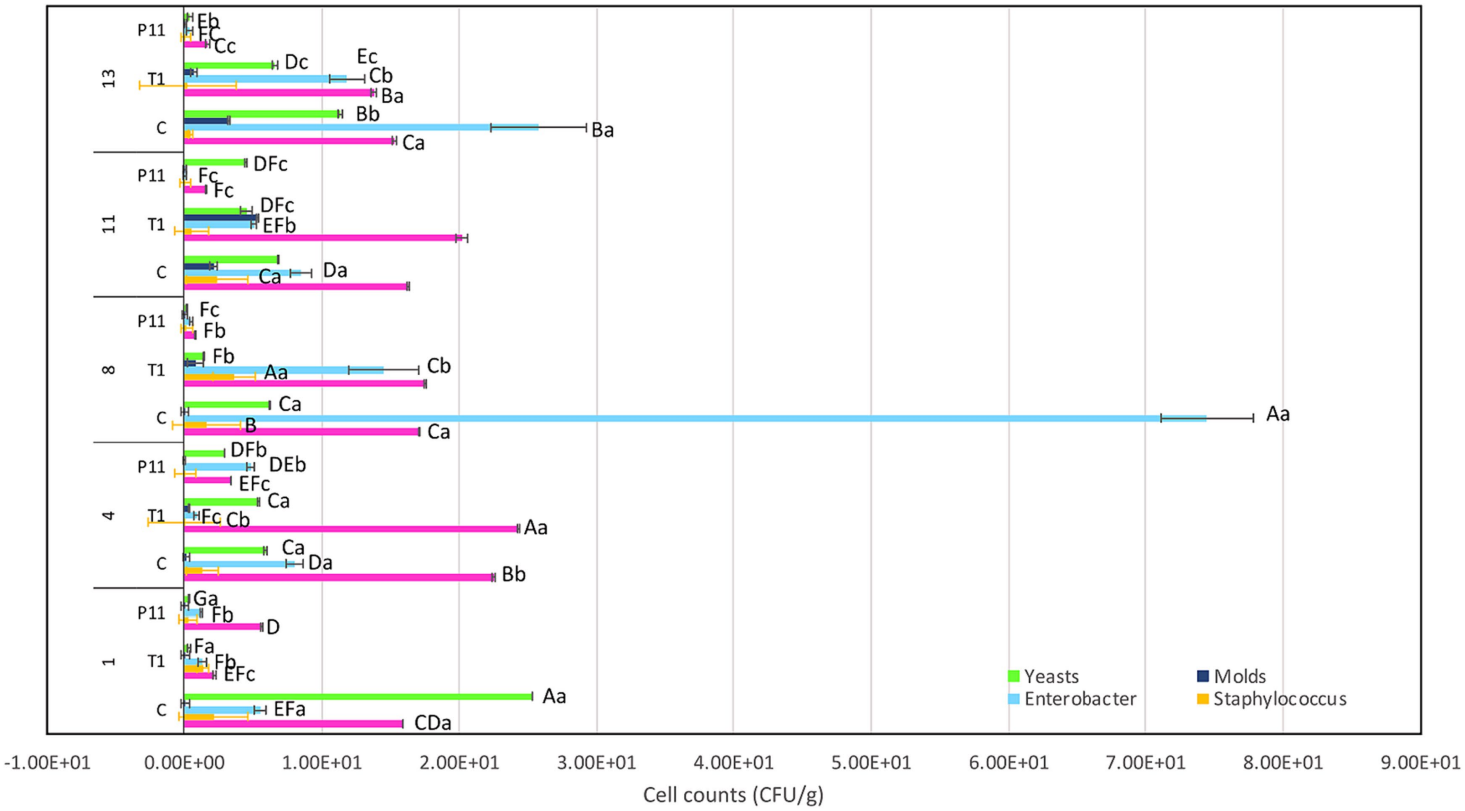
Effect of P11 formulation on target microorganisms associated with avocado fruits during storage. Bars are the means ± standard error. Values with different letters are significantly different *p* < 0.05. Capital letters show the difference between the treatments and pathogen (LSD with Bonferroni correction); small letters show the differences within the storage time (Tukey test). C: control, untreated; T1: commercial disinfectant; P11: (1 x MIC) UTNGt21O + UTNGt28 (1:3, v/v); UTNGt21O: metabiotics from *W. cibaria* strain UTNGt21O; UTNGt28: metabiotics from *L. lactis* strain UTNGt28.

The exocarp is a crucial barrier system for maintaining physical and chemical fruit integrity because it can block the passage of potentially harmful environmental elements, such as invasive microbes. The assessment of the phenotypic characteristics of avocado fruits during storage showed that, in comparison to the untreated (C) and commercial decontaminant solution (T1) counterparts, the application of P11 formulation delayed fruit damage by approximately 1 week (Supplementary Table S2). We noticed that by days 7–8, samples treated with T1 and untreated samples displayed noticeably larger black spots on their exocarp and mesocarp (pulp), whereas P11 had slower black spot formation on both the exocarp and mesocarp (Table 1). It’s also possible that the P11 formulation prevents microorganism colonization while maintaining the fruit’s qualitative (physiological, functional) and subjective (aspect) qualities because there was no visible contamination in the pulp. In a supplementary study, we examined the effect of the PT11 formulation (which contains Tween 20) on avocado fruits. However, we found that this formulation slows down the complete drying of the fruit, meaning that while it exhibited highly effective inhibitory action *in vitro*, its *ex vitro* effects were not as strong. It is crucial to choose a formulation that dries quickly on the fruit’s surface after submersion. Due to its high lipid and moisture content, low carbohydrate content, and non-acidic pH, previous studies have shown that avocados can be a good growth medium for pathogens (FDA, 2018). Fungal infection, physical harm, lenticel breakdown, water loss, cold injury, or a combination of these causes may all contribute to this disease (Hernández et al., 2023). Previous investigations carried out in different climatic zones in Peru indicate that black spot symptoms and nearby green tissue on the same fruit are likely related to physical damage, fungal invasion, and chilling injury (Everett et al., 2015). Nonetheless, a recent research study on Hass avocados indicated that black spot is a physiological disease caused by the sort of oxidative stress that develops after the fruit is stored for an extended period (Lindh et al., 2021). In our study, the fruits at the immature stage did not show any injury, fungi, or visible black spots during the initial experimentation and were stored at room temperature (19 and 21°C). We do not yet know the precise amount of active ingredient that was absorbed by the fruit exocarp, but covering or suspending the fruits in active molecules appears to be a potential strategy for active protection. In a prior study, we demonstrated that dipping tomato fruits in peptides caused a delay in the formation of fungus, indicating that the active peptides on the fruit membrane may be responsible for this action (Tenea and Pozo Delgado, 2019). Even though the microorganism population (total coliforms, *Staphylococcus* spp., *Enterobacter* spp., molds, and yeasts) had not been eliminated by day 13 of storage, within groups, by day 8 of storage, statistically significant differences (*p* < 0.05) in cell counts were observed, with a high amount of *Enterobacter* spp., total coliforms, and yeasts found in control and T1-treated samples (Figure 4). The P11 formulation treatment sensitized microorganism colonization as no increase in cell counts was observed during storage. These findings imply that the active molecules (i.e., active peptides, lipids, glycolipids, acids, and diacetyl) containing LAB extract may form a barrier between the fruit’s surface and the surrounding environment, preventing the growth of microorganisms, minimizing fruit damage, and, ultimately, extending the fruit’s shelf life. These findings correlate with the *in vitro* results, suggesting the effectiveness of the formulation to inhibit microorganism growth in avocado fruits. Thus, the research and development of novel bio-protector prototypes based on molecules from different LAB species to avoid the downsides of conventional chemical treatments might be a better solution to maintain the quality of fruits with thin exocarps. It will be interesting to consider the concentration of active ingredients absorbed by the fruit exocarp and how this, along with the plant defense mechanism, contributes to the overall inhibitory action against harmful microorganisms.

**Table 1:** Summary of the subjective attributes (color, aspect) of avocado fruits.

**Treatments / Time storage**	**Day 0–4**			**Day 5–10**			**Day 11–13**		
	**Peel aspect and tonality/Pulp aspect**			**Peel aspect and tonality/Pulp aspect**			**Peel aspect and tonality/Pulp aspect**		
C	Shiny exocarp, firm and green/hard			Soft, larger black spots on the fruit surface/small dark spots on the pulp			Soft, larger dark spots on the fruit surface/shriveling/larger dark spots on the pulp		
T1	Shiny exocarp, firm and green/hard			Soft, larger black spots on the fruit surface/small dark spots on the pulp			Soft, larger dark spots on the fruit surface/shriveling/larger dark spots on the pulp		
P11	Shiny exocarp, firm and green/hard			Soft, small black spots on the fruit surface/dull green/soft, intact pulp			Soft, small black spots on the fruit surface/dull green/soft, intact pulp		
Peel (exocarp)	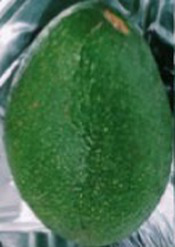	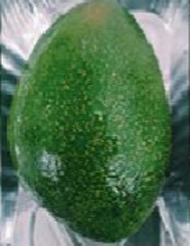	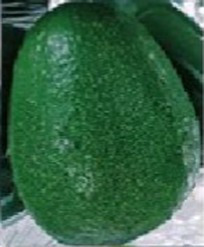	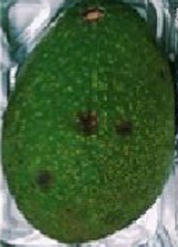	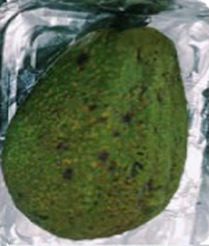	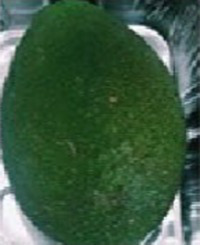	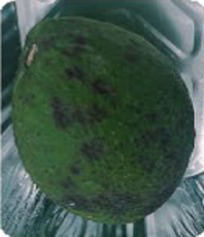	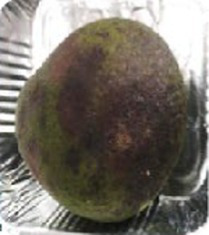	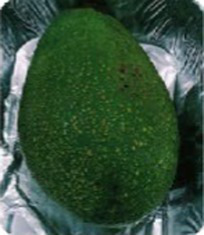
Pulp (mesocarp)	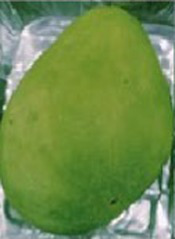	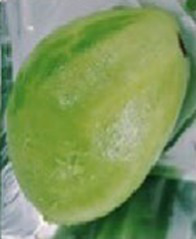	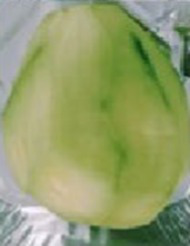	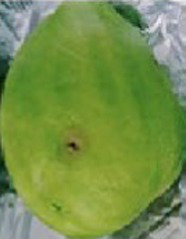	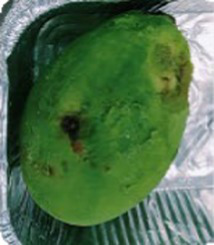	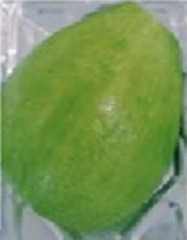	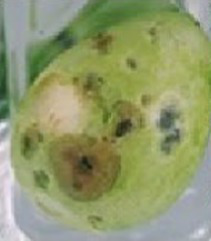	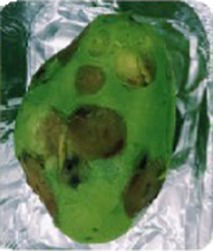	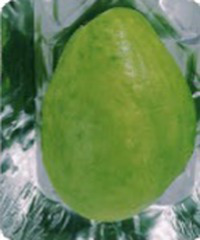
	C	T1	P11	C	T1	P11	C	T1	P11

C: control, untreated; T1: commercial disinfectant; P11: (1 x MIC) UTNGt21O + UTNGt28 (1:3, v/v); UTNGt21O: metabiotics from *W. cibaria* strain UTNGt21O; UTNGt28: metabiotics from *L. lactis* strain UTNGt28.

### P11 treatment does not affect the physicochemical and functional attributes of avocados during storage

**Figure 5:**
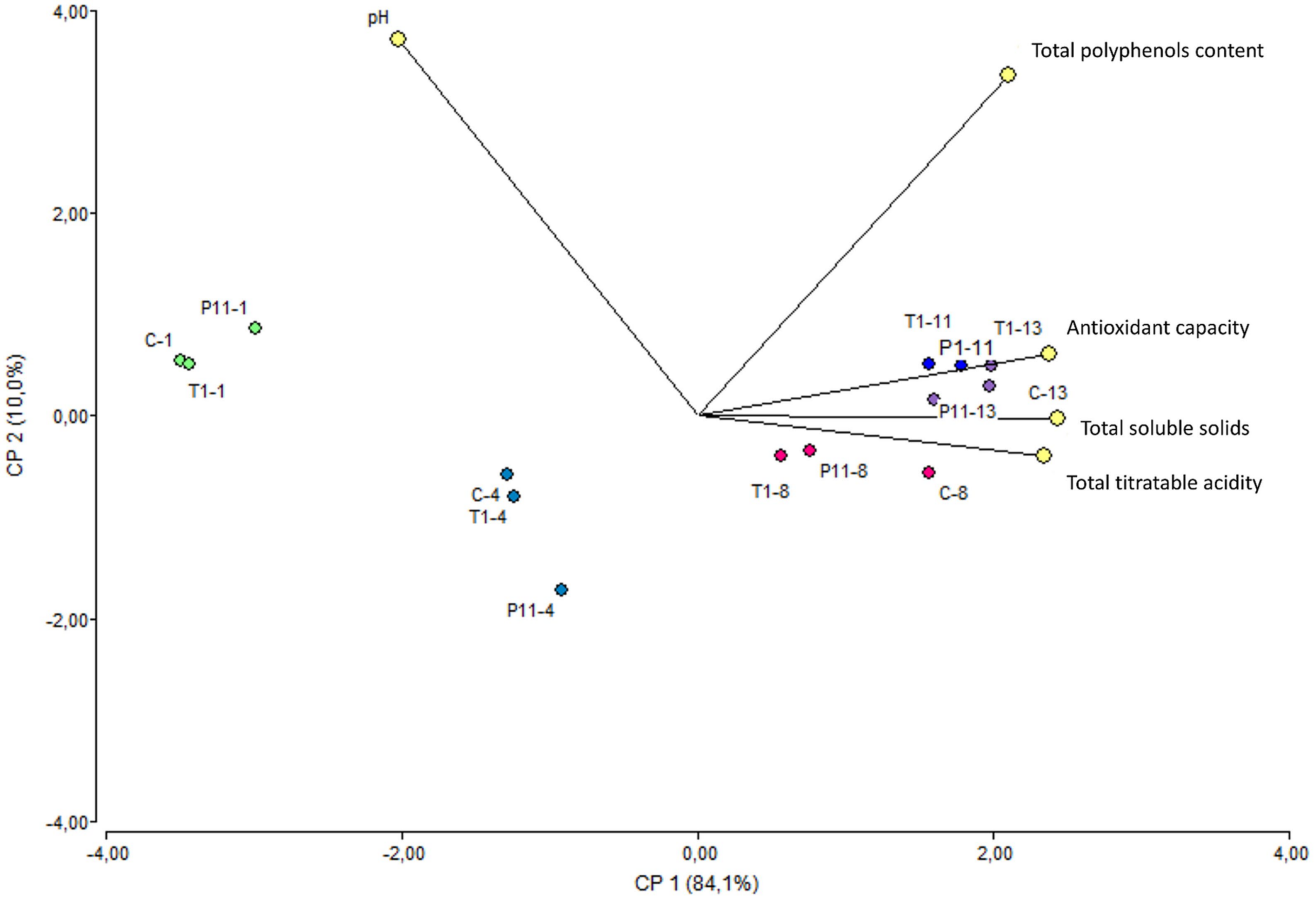
Biplot PCA analysis of the five variables of treated and untreated avocados during storage. The colors marked the close-related samples registered for each variable on days 1, 4, 8, 11, and 13 of storage. C: control, untreated; T1: commercial disinfectant; P11: (1 x MIC) UTNGt21O + UTNGt28 (1:3, v/v); UTNGt21O: metabiotics from *W. cibaria* strain UTNGt21O; UTNGt28: metabiotics from *L. lactis* strain UTNGt28.

Avocado Fuerte fruit has a pH near neutrality (Astudillo-Ordóñez and Rodríguez, 2018). A statistically significant difference (*p* < 0.05) was observed for the pH variable over the storage time, with a small reduction regardless of the treatment (Supplementary Table S3). According to early research, avocado pH values tend to change when they are exposed to cold chain treatments; otherwise, the pH remains near neutrality throughout the ripening process, which is in agreement with our study (Kassim and Workneh, 2020). Similarly, a small increase in total titratable acidity was noted regardless of the treatment. Previous research monitoring the physical and chemical parameters in the Hass variety stored at room temperature reported that the transport of organic acids from intercellular sites to the avocado puree was responsible for the pH drop (Jacobo-Velázquez and Hernández-Brenes, 2011). Recent studies have shown that the deterioration of avocados is linked to an increase in total titratable acidity, which is caused by an increase in free fatty acid concentration brought on by triglyceride lipolysis (Rico-Londoño et al., 2021). Throughout the storage period, avocados exposed to the T1 and P11 treatments as well as C (control) showed a similar trend of increasing total soluble solids by day 8 (Supplementary Table S3). Based on previous research, avocados produce a significant amount of cellulose during ripening, which represents an increase in glucose concentration (Astudillo-Ordóñez and Rodríguez, 2018); this might explain the increase in sugar content during storage. In addition, the enhanced hydrolysis of stored carbohydrates into soluble sugars within the avocado fruit under ambient storage conditions has been found to be influenced by elevated temperature and decreased humidity (Kassim and Workneh, 2020). However, in contrast to earlier research on the Hass variety, which found that fruits stored at room temperature with various packing treatments had significantly increased total soluble solids over storage time (Aguirre-Joya et al., 2017), the total soluble solids values in this study were maintained following treatment with P11 formulation. This may be related to the slower senescence and ripening to a lesser extent. This correlates with the phenotypic observations of delayed damage in the P11-treated fruits. The pulp of P11-treated fruits does not show any black spots as observed in the final state of control and T1-treated fruits. A similar trend in increasing the total polyphenol content and antioxidant capacity values over time was observed in all treatments (Supplementary Table S3). PCA analysis conducted on the five variables showed a clear separation according to the storage time rather than treatment (Figure 5). The variable F1 explained 84.1% of the total variance, while F2 explained 10.0%. The results showed that on days 1 and 4 of storage, the treated and untreated fruits were characterized by greater pH, while on days 8, 11, and 13, they showed greater levels of total soluble solids and antioxidant capacity. In addition, the total polyphenol content vector forms an angle of approximately 90° between the other vectors, meaning that is an independent vector, or it has a weak relationship with the other variables. The total titratable acidity and total soluble solids variables showed the highest Pearson correlation, with a value of 0.97, followed by antioxidant capacity and total soluble solids, with a value of 0.93, suggesting that with increasing total titratable acidity, total soluble solids increased. Additionally, pH and total soluble solids and pH and antioxidant capacity have the strongest correlations, with values of −0.78 and −0.75, respectively, indicating an inverse relationship between these variables. According to Rodríguez-Carpena et al. (2011), ripening has a significant impact on the amount of antioxidant capacity found in the avocado pulp of the Fuerte variety. This might support our findings, showing higher values at the fruit ripening stage. Based on this study, treated avocados with P11 formulation illustrate the benefit of pre-treatment in maintaining the quality of avocado fruit during storage at room temperature.

## Conclusion

In this research, we systematically evaluated the antimicrobial activity of several PPE-based formulations toward *Staphylococcus* strains isolated from the Fuerte variety of avocado and studied the mode of action of the most efficient combination, both *in vitro* and *ex vitro*. It was found that the P11 formulation diminished the growth of both *Staphylococcus* strains by impairing the integrity of the cell membrane, inducing cytoplasm molecule content leakage, protein profile changes, and, finally, bacterial death. Pre-treatment of avocado fruits at the immature stage with the P11 formulation sensitizes microorganism colonization on the fruit surface, minimizing damage to the fruit, whereas quality attributes are preserved. These results suggest its considerable potential for reducing bacterial contamination after harvest and serve as a basis for further research in developing novel fruit bio-protectors based on metabolites obtained from different LAB species. Research on formulation stability (temperature, pH) during storage is ongoing. Finally, the application of metabiotics coating was beneficial in preserving the postharvest features of avocados, as evidenced by the delay in fruit deterioration, increased shelf-life, and retained fruit quality.

## Data availability statement

The original contributions presented in the study are included in the article/Supplementary material, further inquiries can be directed to the corresponding author.

## Author contributions

GNT: Visualization, Validation, Writing – review & editing, Writing – original draft, Supervision, Software, Resources, Project administration, Methodology, Investigation, Funding acquisition, Formal analysis, Data curation, Conceptualization. EA: Writing – review & editing, Software, Investigation, Formal analysis. VC: Writing – review & editing, Investigation. JH: Writing – review & editing, Investigation.

## References

[ref1] Agriopoulou S. Stamatelopoulou E. Sachadyn-Król M. Varzakas T. (2020). Lactic acid Bacteria as antibacterial agents to extend the shelf life of fresh and minimally processed fruits and vegetables: quality and safety aspects. Microorganisms 8:952. doi: 10.3390/microorganisms8060952, PMID: 32599824 PMC7356186

[ref2] Aguirre-Joya J. A. Ventura-Sobrevilla J. Martinez-Vazquez G. Ruelas-Chacon X. Rojas R. Rodriguez-Herrera R.. (2017). Effects of a natural bioactive coating on the quality and shelf life prolongation at different storage conditions of avocado (*Persea americana* mill.) cv. Hass. Food Packag. Shelf Life. 14, 102–107. doi: 10.1016/j.fpsl.2017.09.003

[ref3] Alakomi H. L. Paananen A. Suihko M. L. Helander I. M. Saarela M. (2006). Weakening effect of cell permeabilizers on gram-negative bacteria causing biodeterioration. Appl. Environ. Microbiol. 72, 4695–4703. doi: 10.1128/AEM.00142-06, PMID: 16820461 PMC1489302

[ref4] Aliero A. A. Turba F. Y. Bagudo A. I. Folake A. A. Manga S. S. (2022). Antibiotics resistant pattern of Bacteria isolated from spoiled avocado fruit sold in Sokoto Metropolis. Borneo J. Pharm. 5, 367–374. doi: 10.33084/bjop.v5i4.3405

[ref5] Álvarez Flores J. J. Vite Cevallos H. Garzón Montealegre V. J. Carvajal Romero H. (2021). Análisis de la producción de aguacate en el Ecuador y su exportación a mercados internacionales en el periodo 2008 al 2018. REMCA 4, 164–172.

[ref6] Angamarca E. Castillejo P. Tenea G. N. (2023). Microbiota and its antibiotic resistance profile in avocado Guatemalan fruits (*Persea nubigena var. guatemalensis*) sold at retail markets of Ibarra city, northern Ecuador. Front. Microbiol. 14:1228079. doi: 10.3389/fmicb.2023.1228079, PMID: 37744909 PMC10513466

[ref7] AOAC (2003) Official method of analysis, 17th edn. Association of Official Analytical Chemistry, Washington, 24.

[ref8] Astudillo-Ordóñez C. E. Rodríguez P. (2018). Physicochemical parameters of avocado *Persea americana* mill. Cv. Hass (Lauraceae) grown in Antioquia (Colombia) for export. Corpoica Ciencia Tecnol. Agropecuaria 19, 393–402. doi: 10.21930/rcta.vol19_num2_art:694

[ref9] Bahrami A. Delshadi R. Assadpour E. Jafari S. M. Williams L. (2020). Antimicrobial-loaded nanocarriers for food packaging applications. Adv. Colloid Interf. Sci. 278:102140. doi: 10.1016/j.cis.2020.102140, PMID: 32171115

[ref10] Barbosa A. A. T. Silva de Araújo H. G. Matos P. N. Carnelossi M. A. G. Almeida de Castro A. (2013). Effects of nisin-incorporated films on the microbiological and physicochemical quality of minimally processed mangoes. Int. J. Food Microbiol. 164, 135–140. doi: 10.1016/j.ijfoodmicro.2013.04.004, PMID: 23673058

[ref11] Biswas I. Das Mohapatra P. K. (2023). Recent advancement in metabiotics: a consortium with bioactive molecules after fermentation by probiotic bacteria with multidisciplinary application potential and future solution in health sector. Biores. Technol. Rep. 23:101583. doi: 10.1016/j.biteb.2023.101583

[ref12] Christensen I. B. Vedel C. Clausen M. L. Kjærulff S. Agner T. Nielsen D. S. (2021). Targeted screening of lactic acid Bacteria with antibacterial activity toward *Staphylococcus aureus* clonal complex type 1 associated with atopic dermatitis. Front. Microbiol. 12:733847. doi: 10.3389/fmicb.2021.733847, PMID: 34603263 PMC8486014

[ref13] Everett K.R. Pushparajah I.P.S. Woolf A.B. Burdon J.N. Escobedo V. Vasquez K. (2015). Investigation of the cause of ‘black spot’ disorder of avocado fruit in Peru. Proceedings postharvest and processing. VIII Congraso Mundial de la Palta. Available at: https://www.avocadosource.com/wac8/section_05/everettkerri2015b.pdf (Accessed September 14, 2023)

[ref14] Fan S. Qi Y. Shi L. Giovani M. Zaki N. A. A. Guo S.. (2022). Screening of phenolic compounds in rejected avocado and determination of their antioxidant potential. PRO 10:1747. doi: 10.3390/pr10091747

[ref15] FAO 21.CFR.172.120. (2020). Code of federal Regulation-title 21. Available at: https://www.accessdata.fda.gov/scripts/cdrh/cfdocs/cfcfr/CFRSearch.cfm?fr=172.120&SearchTerm=calciumdisodiumedta (Accessed September, 10, 2023).

[ref16] FDA. (2018). FY 2014–2016 Microbiological Sampling Assignment - Summary Report: Whole Fresh Avocados. Available at: https://www.fda.gov/media/119969/download (Accessed November 1, 2023).

[ref17] FDA American Food Drug Administration. *Carnobacterium divergens* M35 Culture for Use as a bio-preservative in fish product. (2016). Available at: https://www.fda.gov/media/165250/download (Accessed September 20, 2023)

[ref18] García-Frutos R. Martínez-Chávez L. Cabrera-Díaz E. Gutiérrez-González P. Montañez-Soto J. L. Varela-Hernández J. J.. (2020). *Salmonella, listeria monocytogenes*, and indicator microorganisms on Hass avocados sold at retail markets in Guadalajara. Mexico. J. Food Prot. 83, 75–81. doi: 10.4315/0362-028x.jfp-19-273, PMID: 31851548

[ref19] Ge J. Sun Y. Xin X. Wang Y. Ping W. (2016). Purification and partial characterization of a novel bacteriocin synthesized by *Lactobacillus paracasei* HD1–7 isolated from Chinese sauerkraut juice. Sci. Rep. 6:19366. doi: 10.1038/srep19366, PMID: 26763314 PMC4725913

[ref20] Ghanbari M. Jami M. Kneifel W. Doming K. J. (2013). Antimicrobial activity and partial characterization of bacteriocins produced by lactobacilli isolated from sturgeon fish. Food Control 32, 379–385. doi: 10.1016/j.foodcont.2012.12.024

[ref21] Gomez-Lopez A. Aberkane A. Petrikkou E. Mellado E. Rodriguez-Tudela J. L. Cuenca-Estrella M. (2006). Analysis of the influence of tween concentration, inoculum size, assay medium, and reading time on susceptibility testing of *Aspergillus* spp. J. Clin. Microbiol. 43, 1251–1255. doi: 10.1128/JCM.43.3.1251-1255.2005, PMID: 15750092 PMC1081276

[ref23] Hernández D. García-Pérez O. Perera S. González-Carracedo M. A. Rodríguez-Pérez A. Siverio F. (2023). Fungal pathogens associated with aerial symptoms of avocado (*Persea americana* mill.) in Tenerife (Canary Islands, Spain) focused on species of the family *Botryosphaeriaceae*. Microorganisms 11:585. doi: 10.3390/microorganisms11030585, PMID: 36985159 PMC10058760

[ref24] Islam S. Biswas S. Jabin T. Moniruzzaman M. Biswas J. Uddin M. S.. (2023). Probiotic potential of *Lactobacillus plantarum* DMR14 for preserving and extending shelf life of fruits and fruit juice. Heliyon 9:e17382. doi: 10.1016/j.heliyon.2023.e17382, PMID: 37484375 PMC10361358

[ref25] ISO 6888-1:1999/Amd 2:2018. (n.d.) Microbiology of food and animal feeding stuffs – Horizontal method for the enumeration of coagulase-positive staphylococci (*Staphylococcus aureus* and other species) – Part 1: Technique using Baird-Parker agar medium – Amendment 2: Inclusion of an alternative confirmation test using RPFA stab method. Available at: https://standards.iteh.ai/catalog/standards/sist/d4cf5ef2-7a26-48f3-92fab46a9f160aa9/iso-6888-1-1999-amd-2-2018

[ref26] Jacobo-Velázquez D. A. Hernández-Brenes C. (2011). Sensory shelf-life limiting factor of high hydrostatic pressure processed avocado paste. J. Food Sci. 76, S388–S395. doi: 10.1111/j.1750-3841.2011.02259.x, PMID: 21729075

[ref27] Juda M. Paprota K. Malm A. (2008). EDTA as a potential agent preventing formation of *Staphylococcus epidermidis* biofilm on polichloride vinyl biomaterials. Ann. Agric. Environ. Med. 15, 237–241. PMID: 19118444

[ref28] Kassim A. Workneh T. S. (2020). Influence of postharvest treatments and storage conditions on the quality of Hass avocados. Heliyon 6:e04234. doi: 10.1016/j.heliyon.2020.e04234, PMID: 32642570 PMC7334236

[ref29] Lindh V. Uarrota V. Zulueta C. Alvaro J. E. Valdenegro M. Cuneo I. F.. (2021). Image analysis reveals that lenticel damage does not result in black spot development but enhances dehydration in *Persea americana* mill. cv Hass during prolonged storage. Agronomy 11:1699. doi: 10.3390/agronomy11091699

[ref30] Lyu X. Agar O. T. Barrow C. J. Dunshea F. R. Suleria H. A. R. (2023). Phenolic compounds profiling and their antioxidant capacity in the Peel, pulp, and seed of Australian grown avocado. Antioxidants (Basel). 12:185. doi: 10.3390/antiox12010185, PMID: 36671046 PMC9855119

[ref31] MAGAP. (2021). Principales productos agropecuarios. Available at: http://sipa.agricultura.gob.ec/index.php/platano (Accessed September 26, 2023).

[ref32] Munhuweyi K. Mpai S. Sivakumar D. (2020). Extension of avocado fruit postharvest quality using non-chemical treatments. Agronomy 10:212. doi: 10.3390/agronomy10020212

[ref33] Musa A. Wiman E. Selegård R. Aili D. Bengtsson T. Khalaf H. (2021). Plantaricin NC8 αβ prevents *Staphylococcus aureus*-mediated cytotoxicity and inflammatory responses of human keratinocytes. Sci. Rep. 11:12514. doi: 10.1038/s41598-021-91682-6, PMID: 34131160 PMC8206081

[ref34] Patra P. Roy S. Sarkar S. Mitra S. Pradhan S. Debnath N.. (2015). Damage of lipopolysaccharides in outer cell membrane and production of ROS-mediated stress within bacteria makes nano zinc oxide a bactericidal agent. Appl. Nanosci. 5, 857–866. doi: 10.1007/s13204-014-0389-z

[ref35] Poleatewich A. Backman P. Nolen H. (2023). Evaluation of endospore-forming Bacteria for suppression of postharvest decay of apple fruit. Microorganisms 11:81. doi: 10.3390/microorganisms11010081 PMC9862789 36677372

[ref36] Rico-Londoño J. F. Buitrago-Patiño D. J. Agudelo-Laverde L. M. (2021). Combination of methods as alternative to maintain the physical-chemical properties and microbiological content of Hass avocado pulp during storage. Food Biosci. 44:101372. doi: 10.1016/j.fbio.2021.101372

[ref37] Rodríguez-Carpena J. G. Morcuende D. Andrade M. J. Kylli P. Estevez M. (2011). Avocado (*Persea americana* mill.) phenolics, *in vitro* antioxidant and antimicrobial activities, and inhibition of lipid and protein oxidation in porcine patties. J. Agri. Food Chem. 59, 5625–5635. doi: 10.1021/jf1048832, PMID: 21480593

[ref39] Romanazzi G. Feliziani E. Sivakumar D. (2018). Chitosan, a biopolymer with triple action on postharvest decay of fruit and vegetables: eliciting, antimicrobial and film-forming properties. Front. Microbiol. 9:2745. doi: 10.3389/fmicb.2018.02745, PMID: 30564200 PMC6288236

[ref40] Selegård R. Musa A. Nyström P. Aili D. Bengtsson T. Khalaf H. (2019). Plantaricins markedly enhance the effects of traditional antibiotics against *Staphylococcus epidermidis*. Future Microbiol. 14, 195–205. doi: 10.2217/fmb-2018-0285, PMID: 30648887 PMC6393846

[ref41] Siroli L. Patrignani F. Serrazanetti D. I. Tabanelli G. Montanari C. Gardini F. (2015). Lactic acid bacteria and natural antimicrobials to improve the safety and shelf life of minimally processed sliced apples and lamb’s lettuce. Food Microbiol. 47, 74–84. doi: 10.1016/j.fm.2014.11.008, PMID: 25583340

[ref42] Ssemugabo C. Bradman A. Ssempebwa J. C. Guwatudde D. (2023). Consumer awareness and health risk perceptions of pesticide residues in fruits and vegetables in Kampala metropolitan area in Uganda. Environ. Health. Insights. 17:11786302231184751. doi: 10.1177/11786302231184751, PMID: 37476078 PMC10354737

[ref43] Tenea G. N. Hurtado P. (2021). Next-generation sequencing for whole-genome characterization of *Weissella cibaria* UTNGt21O strain originated from wild *Solanum quitoense* lam. Fruits: an atlas of metabolites with biotechnological significance. Front. Microbiol. 12:675002. doi: 10.3389/fmicb.2021.675002, PMID: 34163450 PMC8215347

[ref44] Tenea G. N. Pozo Delgado T. (2019). Antimicrobial peptides from *Lactobacillus plantarum* UTNGt2 prevent harmful bacteria growth on fresh tomatoes. J. Microbiol. Biotechnol. 29, 1553–1560. doi: 10.4014/jmb.1904.04063, PMID: 31434171

[ref45] Tenea G. N. Reyes P. Molina D. Ortega C. (2023). Pathogenic microorganisms linked to fresh fruits and juices purchased at low-cost markets in Ecuador, potential carriers of antibiotic resistance. Antibiotics 12:236. doi: 10.3390/antibiotics12020236, PMID: 36830147 PMC9952111

[ref46] Terpou A. Papadaki A. Lappa I. K. Kachrimanidou V. Bosnea L. A. Kopsahelis N. (2019). Probiotics in food systems: significance and emerging strategies towards improved viability and delivery of enhanced beneficial value. Nutrients 11:1591. doi: 10.3390/nu11071591, PMID: 31337060 PMC6683253

[ref47] Tesfay S. Z. Magwaza L. S. (2017). Evaluating the efficacy of moringa leaf extract, chitosan and carboxymethyl cellulose as edible coatings for enhancing quality and extending postharvest life of avocado (*Persea americana* mill.) fruit. Food Packag. Shelf Life 11, 40–48. doi: 10.1016/j.fpsl.2016.12.001

[ref48] Tumbarski Y. Nikolova R. Petkova N. Ivanov I. Lante A. (2019). Biopreservation of fresh strawberries by carboxymethyl cellulose edible coatings enriched with a bacteriocin from *Bacillus methylotrophicus* BM47. Food Technol. Biotechnol. 57, 230–237. doi: 10.17113/ftb.57.02.19.6128, PMID: 31537972 PMC6718969

[ref49] Vieco-Saiz N. Belguesmia Y. Raspoet R. Auclair E. Gancel F. Kempf I.. (2019). Benefits and inputs from lactic acid bacteria and their bacteriocins as alternatives to antibiotic growth promoters during food-animal production. Front. Microbiol. 10:57. doi: 10.3389/fmicb.2019.00057, PMID: 30804896 PMC6378274

[ref50] Vieira A. I. Guerreiro A. Antunes M. D. Miguel M. G. Faleiro M. (2019). Edible coatings enriched with essential oils on apples impair the survival of bacterial pathogens through a simulated gastrointestinal system. Food Secur. 8:57. doi: 10.3390/foods8020057, PMID: 30720754 PMC6406970

[ref51] Wang Y. Haqmal M. A. Liang Y. D. Muhammad I. Zhao X. O. Elken E. M.. (2022). Antibacterial activity and cytotoxicity of a novel bacteriocin isolated from *Pseudomonas* sp. strain 166. Microbial Biotechnol 15, 2337–2350. doi: 10.1111/1751-7915.14096, PMID: 35849816 PMC9437881

[ref53] Xue R. Liu Y. Zhang Q. Liang C. Qin H. Liu P.. (2016). Shape changes and interaction mechanism of *Escherichia coli* cells treated with sericin and use of a sericin-based hydrogel for wound healing. Appl. Environ. Microbiol. 82, 4663–4672. doi: 10.1128/AEM.00643-16, PMID: 27235427 PMC4984295

[ref54] Yasir M. Dutta D. Willcox M. D. P. (2019). Mode of action of the antimicrobial peptide Mel4 is independent of *Staphylococcus aureus* cell membrane permeability. PLoS One 14:e0215703. doi: 10.1371/journal.pone.0215703, PMID: 31356627 PMC6663011

[ref55] Zhu C. Zhao Y. Zhao X. Liu S. Xia X. Zhang S.. (2022). The antimicrobial peptide MPX can kill *Staphylococcus aureus*, reduce biofilm formation, and effectively treat bacterial skin infections in mice. Front. Vet. Sci. 9:819921. doi: 10.3389/fvets.2022.819921, PMID: 35425831 PMC9002018

