# Supplementary material for: Preventing microbe colonization on avocado (Persea nubigena var. guatemalensis) through metabiotic treatment, a promising postharvest safety improvement
Source: Front Microbiol. 2024 Mar 13;15:1344735. doi: 10.3389/fmicb.2024.1344735 (PMC10965548; doi:10.3389/fmicb.2024.1344735)
Supplement: Supplementary file 1 [file Data_Sheet_1.docx]

Supplementary Material

**Supplementary Table 1.** Descriptions of formulation compositions.

| **Code PPE** | **Description (concentration/Lactic acid bacteria producer strain code)** |
| --- | --- |
| P1 | (1 x MIC) UTNGt5 |
| P2 | (1 x MIC) UTNGt21O |
| P3 | (1 x MIC) UTNGt28 |
| P4 | (1 x MIC) UTNGt5 + UTNGt21O (1:1, v/v) |
| P5 | (1 x MIC) UTNGt5 + UTNGt21O (1:3, v/v) |
| P6 | (1 x MIC) UTNGt5 + UTNGt21O (3:1, v/v) |
| P7 | (1 x MIC) UTNGt5 + UTNGt28 (1:1, v/v) |
| P8 | (1 x MIC) UTNGt5 + UTNGt28 (1:3, v/v) |
| P9 | (1 x MIC) UTNGt5 + UTNGt28 (3:1, v/v) |
| P10 | (1 x MIC) UTNGt21O + UTNGt21O (1:1, v/v) |
| P11 | (1 x MIC) UTNGt21O + UTNGt28 (1:3, v/v) |
| P12 | (1 x MIC) UTNGt21O + UTNGt28 (3:1, v/v) |
| PEF1 | (1 x MIC) UTNGt5 + EDTA (ethylenediaminetetraacetic acid, 0.1mg/mL) |
| PEF2 | (1 x MIC) UTNGt21O + EDTA (ethylenediaminetetraacetic acid, 0.1 mg/mL) |
| PEF3 | (1 x MIC) UTNGt28 + EDTA (ethylenediaminetetraacetic acid, 0.1 mg/mL) |
| PEF4 | (1 x MIC) UTNGt5 + UTNGt21O (1:1, v/v) + EDTA (ethylenediaminetetraacetic acid, 0.1 mg/mL) |
| PEF5 | (1 x MIC) UTNGt5 + UTNGt21O (1:3, v/v) + EDTA (ethylenediaminetetraacetic acid, 0.1 mg/mL)\ |
| PEF6 | (1 x MIC) UTNGt5 + UTNGt21O (3:1, v/v) + EDTA (ethylenediaminetetraacetic acid, 0.1 mg/mL) |
| PEF7 | (1 x MIC) UTNGt5 + UTNGt28 (1:1, v/v) + EDTA (ethylenediaminetetraacetic acid, 0.1 mg/mL) |
| PEF8 | (1 x MIC) UTNGt5 + UTNGt28 (1:3, v/v) + EDTA (ethylenediaminetetraacetic acid, 0.1 mg/mL) |
| PEF9 | (1 x MIC) UTNGt5 + UTNGt28 (3:1, v/v) + EDTA (ethylenediaminetetraacetic acid, 0.1 mg/mL) |
| PEF10 | (1 x MIC) UTNGt21O + UTNGt28 (1:1, v/v) + EDTA (ethylenediaminetetraacetic acid, 0.1 mg/mL) |
| PEF11 | (1 x MIC) UTNGt21O + UTNGt28 (1:3, v/v) + EDTA (ethylenediaminetetraacetic acid, 0.1 mg/mL) |
| PEF12 | (1 x MIC) UTNGt21O + UTNGt28 (3:1, v/v) + EDTA (ethylenediaminetetraacetic acid, 0.1 mg/mL) |
| PT1 | (1 x MIC) UTNGt5 + Tween 20 (0.01%) |
| PT2 | (1 x MIC) UTNGt21O + Tween 20 (0.01%) |
| PT3 | (1 x MIC) UTNGt28 + Tween 20 (0.01%) |
| PT4 | (1 x MIC) UTNGt5 + UTNGt21O (1:1, v/v) + Tween 20 (0.01%) |
| PT5 | (1 x MIC) UTNGt5 + UTNGt21O (1:3, v/v) + Tween 20 (0.01%) |
| PT6 | (1 x MIC) UTNGt5 + UTNGt21O (3:1, v/v) + Tween 20 (0.01%) |
| PT7 | (1 x MIC) UTNGt5 + UTNGt28 (1:1, v/v) + Tween 20 (0.01%) |
| PT8 | (1 x MIC) UTNGt5 + UTNGt28 (1:3, v/v) + Tween 20 (0.01%) |
| PT9 | (1 x MIC) UTNGt5 + UTNGt28 (3:1, v/v) + Tween 20 (0.01%) |
| PT10 | (1 x MIC) UTNGt21O + UTNGt28 (1:1, v/v) + Tween 20 (0.01%) |
| PT11 | (1 x MIC) UTNGt21O + UTNGt28 (1:3, v/v) + Tween 20 (0.01%) |
| PT12 | (1 x MIC) UTNGt21O + UTNGt28 (3:1, v/v) + Tween 20 (0.01%) |
| CEF | EDTA (ethylenediaminetetraacetic acid, 0.1 mg/mL) |
| CT | Tween 20 (0.01%) |

Legend: UTNGt5: metabiotics from *L. plantarum* strain UTNGt5; UTNGt21O: metabiotics from *W. cibaria* strain UTNGt21O; UTNGt28: metabiotics from *L. lactis* strain UTNGt28; MIC- minimum inhibitory concentration.

**Supplementary Table 2**. Subjective attributes (color, aspect) of avocado fruits during storage.

| **Phenotypic (aspect) evaluation of avocado fruits during storage** | | | | | | | | |
| --- | --- | --- | --- | --- | --- | --- | --- | --- |
| **Treatment** | **# Fruit** | Day 0-4 | Day 5-6 | Day 7-8 | Day 9 | Day 10 | Day 11 | Day 12-13 |
| C | 1 | Shiny exocarp, firm and green/ hard | Small black spots on the fruit surface/ dull green/ intact pulp | Soft, larger black spots on the fruit surface/ small dark spots on the pulp | Soft, larger black spots on the fruit surface/ small dark spots on the pulp | Soft, larger black spots on the fruit surface/ small dark spots on the pulp | Soft, larger black spots on the fruit surface / shriveling/ larger dark spots on the pulp | Soft, larger black spots on the fruit surface / shriveling/ larger dark spots on the pulp |
|  | 2 | Shiny exocarp, firm and green/ hard | Small black spots on the fruit surface/ dull green/ intact pulp | Soft, larger black spots on the fruit surface/ small dark spots on the pulp | Soft, larger black spots on the fruit surface/ small dark spots on the pulp | Soft, larger black spots on the fruit surface/ small dark spots on the pulp | Soft, larger black spots on the fruit surface / shriveling/ larger dark spots on the pulp | Soft, larger black spots on the fruit surface / shriveling/ larger dark spots on the pulp |
|  | 3 | Shiny exocarp, firm and green/ hard | Small black spots on the fruit surface/ dull green/ intact pulp | Soft, larger black spots on the fruit surface/ small dark spots on the pulp | Soft, larger black spots on the fruit surface/ small dark spots on the pulp | Soft, larger black spots on the fruit surface/ small dark spots on the pulp | Soft, larger black spots on the fruit surface / shriveling/ larger dark spots on the pulp | Soft, larger black spots on the fruit surface / shriveling/ larger dark spots on the pulp |
|  | 4 | Shiny exocarp, firm and green/ hard | Small black spots on the fruit surface/ dull green/ intact pulp | Soft, larger black spots on the fruit surface/ small dark spots on the pulp | Soft, larger black spots on the fruit surface/ small dark spots on the pulp | Soft, larger black spots on the fruit surface/ small dark spots on the pulp | Soft, larger black spots on the fruit surface / shriveling/ larger dark spots on the pulp | Soft, larger black spots on the fruit surface / shriveling/ larger dark spots on the pulp |
|  | 5 | Shiny exocarp, firm and green/ hard | Small black spots on the fruit surface/ dull green/ intact pulp | Soft, larger black spots on the fruit surface/ small dark spots on the pulp | Soft, larger black spots on the fruit surface/ small dark spots on the pulp | Soft, larger black spots on the fruit surface/ small dark spots on the pulp | Soft, larger black spots on the fruit surface / shriveling/ larger dark spots on the pulp | Soft, larger black spots on the fruit surface / shriveling/ larger dark spots on the pulp |
| T1 | 1 | Shiny exocarp, firm and green/ hard | Small black spots on the fruit surface/ dull green/ intact pulp | Soft, larger black spots on the fruit surface/ small dark spots on the pulp | Soft, larger black spots on the fruit surface/ small dark spots on the pulp | Soft, larger black spots on the fruit surface / shriveling/ larger dark spots on the pulp | Soft, larger black spots on the fruit surface / shriveling/ larger dark spots on the pulp | Soft, larger black spots on the fruit surface / shriveling/ larger dark spots on the pulp |
|  | 2 | Shiny exocarp, firm and green | Small black spots on the fruit surface/ dull green/ intact pulp | Soft, larger black spots on the fruit surface/ small dark spots on the pulp | Soft, larger black spots on the fruit surface/ small dark spots on the pulp | Soft, larger black spots on the fruit surface / shriveling/ larger dark spots on the pulp | Soft, larger black spots on the fruit surface / shriveling/ larger dark spots on the pulp | Soft, larger black spots on the fruit surface / shriveling/ larger dark spots on the pulp |
|  | 3 | Shiny exocarp, firm and green/ hard | Small black spots on the fruit surface/ dull green/ intact pulp | Soft, larger black spots on the fruit surface/ small dark spots on the pulp | Soft, larger black spots on the fruit surface/ small dark spots on the pulp | Soft, larger black spots on the fruit surface / shriveling/ larger dark spots on the pulp | Soft, larger black spots on the fruit surface / shriveling/ larger dark spots on the pulp | Soft, larger black spots on the fruit surface / shriveling/ larger dark spots on the pulp |
|  | 4 | Shiny exocarp, firm and green/ hard | Small black spots on the fruit surface/ dull green/ intact pulp | Soft, larger black spots on the fruit surface/ small dark spots on the pulp | Soft, larger black spots on the fruit surface/ small dark spots on the pulp | Soft, larger black spots on the fruit surface / shriveling/ larger dark spots on the pulp | Soft, larger black spots on the fruit surface / shriveling/ larger dark spots on the pulp | Soft, larger black spots on the fruit surface / shriveling/ larger dark spots on the pulp |
|  | 5 | Shiny exocarp, firm and green/ hard | Small black spots on the fruit surface/ dull green/ intact pulp | Soft, larger black spots on the fruit surface/ small dark spots on the pulp | Soft, larger black spots on the fruit surface/ small dark spots on the pulp | Soft, larger black spots on the fruit surface / shriveling/ larger dark spots on the pulp | Soft, larger black spots on the fruit surface / shriveling/ larger dark spots on the pulp | Soft, larger black spots on the fruit surface / shriveling/ larger dark spots on the pulp |
| P11 | 1 | Shiny exocarp, firm and green/ hard | Small black spots on the fruit surface/ dull green/ intact pulp | Small black spots on the fruit surface/ dull green/ intact pulp | Small black spots on the fruit surface/ dull green/ intact pulp | Soft, small black spots on the fruit surface/ dull green/ intact pulp | Soft, small black spots on the fruit surface/ dull green/ intact pulp | Soft, small black spots on the fruit surface/ dull green/ intact pulp |
|  | 2 | Shiny exocarp, firm and green/ hard | Small black spots on the fruit surface/ dull green/ intact pulp | Small black spots on the fruit surface/ dull green/ intact pulp | Small black spots on the fruit surface/ dull green/ intact pulp | Soft, small black spots on the fruit surface/ dull green/ intact pulp | Soft, small black spots on the fruit surface/ dull green/ intact pulp | Soft, small black spots on the fruit surface/ dull green/ intact pulp |
|  | 3 | Shiny exocarp, firm and green/ hard | Small black spots on the fruit surface/ dull green/ intact pulp | Small black spots on the fruit surface/ dull green/ intact pulp | Small black spots on the fruit surface/ dull green/ intact pulp | Soft, small black spots on the fruit surface/ dull green/ intact pulp | Soft, small black spots on the fruit surface/ dull green/ intact pulp | Soft, small black spots on the fruit surface/ dull green/ intact pulp |
|  | 4 | Shiny exocarp, firm and green/ hard | Small black spots on the fruit surface/ dull green/ intact pulp | Small black spots on the fruit surface/ dull green/ intact pulp | Small black spots on the fruit surface/ dull green/ intact pulp | Soft, small black spots on the fruit surface/ dull green/ intact pulp | Soft, small black spots on the fruit surface/ dull green/ intact pulp | Soft, small black spots on the fruit surface/ dull green/ intact pulp |
|  | 5 | Shiny exocarp, firm and green/ hard | Small black spots on the fruit surface/ dull green/ intact pulp | Small black spots on the fruit surface/ dull green/ intact pulp | Small black spots on the fruit surface/ dull green/ intact pulp | Soft, small black spots on the fruit surface/ dull green/ intact pulp | Soft, small black spots on the fruit surface/ dull green/ intact pulp | Soft, small black spots on the fruit surface/ dull green/ intact pulp |

Legend: C: avocado fruits washed with distillate water, (Control); T1: avocado fruits treated with commercial disinfectant; P11: avocado fruits treated with (1 x MIC) UTNGt21O + UTNGt28 (1:3, v/v). UTNGt21O: metabiotics from *W. cibaria* strain UTNGt21O; UTNGt28: metabiotics from *L. lactis* strain UTNGt28.

**Supplementary Table 3.** Changes in quality attributes in treated and untreated avocados during storage.

| **Treatments** | **Storage time (day)** | **pH** | **Total soluble solids** | **Total titratable acidity** | **Antioxidant capacity** | **Total polyphenol content** |
| --- | --- | --- | --- | --- | --- | --- |
| C | 1 | 7.00 ± 0.11^ab^ | 0.50 ± 0.00^e^ | 0.016 ± 0.02^de^ | 428.85 ± 0.00^f^ | 69.32 ± 0.01^ef^ |
| T1 |  | 7.13 ± 0.02^a^ | 0.53 ± 0.06^e^ | 0.015 ± 0.00^e^ | 396.53 ± 0.04^f^ | 70.92 ± 0.02^ef^ |
| P11 |  | 7.03 ± 0.07^ab^ | 0.51± 0.00^e^ | 0.015 ± 0.00^e^ | 417.74 ± 0.04^f^ | 62.17 ± 0.03^f^ |
| C | 4 | 6.76 ± 0.44^ab^ | 1.37 ± 0.88^cde^ | 0.035 ± 0.29^abcde^ | 1006.83 ± 0.02^abcd^ | 104.63 ± 0.03^e^ |
| T1 |  | 6.83 ± 0.29^ab^ | 1.42 ± 0.78^bcde^ | 0.028 ± 0.17^bcde^ | 973.70 ± 0.01^cd^ | 104.06 ± 0.03^e^ |
| P11 |  | 6.43 ± 0.20^b^ | 1.34 ± 0.75^de^ | 0.028 ± 0.20^cde^ | 786.22 ± 0.04^e^ | 102.82 ± 0.00^e^ |
| C | 8 | 6.54 ± 0.29^ab^ | 2.82 ± 0.25^a^ | 0.063 ± 0.15^a^ | 1158.14 ± 0.03^a^ | 185.95 ± 0.11^bcd^ |
| T1 |  | 6.65 ± 0.26^ab^ | 2.63 ± 0.23^abcd^ | 0.054 ± 0.02^abc^ | 1130.46 ± 0.03^ab^ | 197.73 ± 0.07^abc^ |
| P11 |  | 6.62 ± 0.15^ab^ | 2.57 ± 0.38^abcd^ | 0.047 ± 0.11^abc^ | 1123.39 ± 0.04^abc^ | 154.45 ± 0.03^d^ |
| C | 11 | 6.59 ± 0.24^ab^ | 2.72 ± 0.41^abcd^ | 0.047 ± 0.13^abc^ | 1005.01 ± 0.01^abcd^ | 199.04 ± 0.06^abc^ |
| T1 |  | 6.46 ± 0.07^b^ | 2.63 ± 0.29^abcd^ | 0.050 ± 0.03^abc^ | 1016.53 ± 0.01^abcd^ | 187.00 ± 0.02^bcd^ |
| P11 |  | 6.69 ± 0.11^ab^ | 2.74 ± 0.47^abc^ | 0.058 ± 0.15^ab^ | 1127.64 ± 0.05^abc^ | 168.39 ± 0.04^cd^ |
| C | 13 | 6.53 ± 0.03^ab^ | 2.57 ± 0.64^abcd^ | 0.048 ± 0.04^abc^ | 987.23 ± 0.03^bcd^ | 210.80 ±0.05^ab^ |
| T1 |  | 6.54 ± 0.27^ab^ | 2.76 ± 0.19^abc^ | 0.051 ± 0.16^abc^ | 955.92 ± 0.03^d^ | 233.52 ± 0.15^a^ |
| P11 |  | 6.56 ± 0.17^ab^ | 2.79 ± 0.27^ab^ | 0.045 ± 0.03^abcd^ | 1015.31 ± 0.02^abcd^ | 196.68 ± 0.04^abc^ |

Means within a column followed by the same letter/s are not significantly different from each other, according to Tukey (*p* < 0.001). Legend: C: control, no treated; T1: commercial disinfectant; P11- (1 x MIC) UTNGt21O + UTNGt28 (1:3, v/v); UTNGt21O: metabiotics from *W. cibaria* strain UTNGt21O; UTNGt28: metabiotics from *L. lactis* strain UTNGt28.

**Supplementary Figure 1**. Inhibition zones formed by P11, PEF11, P2, and P3 against A) *Staphylococcus* clone FFCShyA2 and B) *Staphylococcus* clone FFCShyA4. Legend: R1-R3: repetitions, C-negative control (no formulation). P2- (1 x MIC) UTNGt21O; P3: (1 x MIC) UTNGt28; P11-(1 x MIC) UTNGt21O + UTNGt28 (1:3, v/v); PEF11-(1 x MIC) UTNGt21O + UTNGt28 (1:3, v/v) + EDTA (0.1 mg/mL); UTNGt21O: metabiotics extract from *W. cibaria* strain UTNGt21O; UTNGt28: metabiotics extract from *L. lactis* strain UTNGt28.

A).

B).
